# Novel H-Bonded Synthons in Copper Supramolecular Frameworks with Aminoethylpiperazine-Based Ligands. Synthesis, Structure and Catalytic Activity

**DOI:** 10.3390/ma13235435

**Published:** 2020-11-29

**Authors:** Oksana V. Nesterova, Armando J. L. Pombeiro, Dmytro S. Nesterov

**Affiliations:** 1 Centro de Química Estrutural, Instituto Superior Técnico, Universidade de Lisboa, Av. Rovisco Pais, 1049-001 Lisboa, Portugal; oksana.nesterova@tecnico.ulisboa.pt (O.V.N.); pombeiro@tecnico.ulisboa.pt (A.J.L.P.); 2 Research Institute of Chemistry, Peoples’ Friendship University of Russia (RUDN University), 6 Miklukho-Maklaya st., 117198 Moscow, Russia

**Keywords:** copper complexes, Schiff bases, supramolecular polymers, DFT calculations, exchange couplings, catalysis, alkane functionalization, C–H bonds, amidation

## Abstract

New Schiff base complexes [Cu_2_(HL^1^)(L^1^)(N_3_)_3_]∙2H_2_O (**1**) and [Cu_2_L^2^(N_3_)_2_]∙H_2_O (**2**) were synthesized. The crystal structures of **1** and **2** were determined by single-crystal X-ray diffraction analysis. The HL^1^ ligand results from the condensation of salicylaldehyde and 1-(2-aminoethyl)piperazine, while a new organic ligand, H_2_L^2^, was formed by the dimerization of HL^1^ via a coupling of two piperazine rings of HL^1^ on a carbon atom coming from DMF solvent. The dinuclear building units in **1** and **2** are linked into complex supramolecular networks through hydrogen and coordination bondings, resulting in 2D and 1D architectures, respectively. Single-point and broken-symmetry DFT calculations disclosed negligible singlet–triplet splittings within the dinuclear copper fragments in **1** and **2**. Catalytic studies showed a remarkable activity of **1** and **2** towards cyclohexane oxidation with H_2_O_2_ in the presence of nitric acid and pyridine as promoters and under mild conditions (yield of products up to 21%). Coordination compound **1** also acts as an active catalyst in the intermolecular coupling of cyclohexane with benzamide using di-tert-butyl peroxide (*^t^*BuOO*^t^*Bu) as a terminal oxidant. Conversion of benzamide at 55% was observed after 24 h reaction time. By-product patterns and plausible reaction mechanisms are discussed.

## 1. Introduction

Design of functional coordination polymers involving transition metals is a hot topic in modern chemistry due to the wide range of properties exhibited by this class of materials [1,2]. The presence of a regular lattice with controlled intermetallic separations provides a basis for novel magnetic materials [3,4] and creates favourable conditions for catalytic activity [5,6,7]. Porosity is another important feature of coordination polymers and metal–organic frameworks, which enables their applications for gas storage and controlled release, activation of small molecules, drug delivery, and many others [8,9,10]. Activation and subsequent functionalization of alkanes bearing inert sp^3^ C–H bonds towards industrially significant products is one of the fields of relevant importance in modern catalysis [11], and therefore, the development of efficient new catalytic systems is a topic of current attention [5,12,13,14]. Moreover, design and preparation of simple, cheap, and efficient catalysts based on copper complexes attract special attention [15], being inspired by the natural copper-containing enzymes (methane monooxygenases), which catalyse the oxidation of methane and heavier alkanes at ambient conditions [12].

Among many different ligands that can be used for the preparation of polymeric and polynuclear coordination compounds [5,16,17], the Schiff bases attract a strong continuous interest since they can be easily designed as novel ligand systems through the in situ condensation of a large library of amines and aldehydes [18,19]. Earlier, we reported the iron(III) complex [Fe(HL^1^)Cl_2_(DMF)]Cl∙DMF (where HL^1^ is the Schiff base ligand, resulting from the condensation of salicylic aminoethylpiperazine and aldehyde), which showed a high catalytic activity in cyclohexane oxidation with H_2_O_2_ [20]. Additionally, it was found that the copper complexes [Cu(HL^1^)(NO_3_)(DMF)](NO_3_)∙H_2_O and [Cu(HL^1^)Cl_2_]∙½DMSO with the same ligand, HL^1^, as well as the compound [CuCl_2_L^a^]·DMF, where L^a^ is a product of the condensation of aminoguanidine and 2-pyridinecarbaldehyde, display a prominent behaviour in this catalytic reaction but using a pyridine as promoting agent [21]. Continuing our studies aiming at synthesis and investigation of novel catalytic systems [5,22,23,24,25,26], we prepared the two new complexes [Cu_2_(HL^1^)(L^1^)(N_3_)_3_]∙2H_2_O (**1**) and [Cu_2_L^2^(N_3_)_2_]∙H_2_O (**2**) bearing known HL^1^− (in **1**) and novel H_2_L^2^-derived (in **2**) Schiff base ligands, studied their solid-state crystal structures, analysed the spin density distributions, and tested their catalytic activity in the reactions of amidation of cyclohexane with benzamide using *^t^*BuOO*^t^*Bu as oxidant and oxidation of cyclohexane with hydrogen peroxide.

## 2. Materials and Methods

All the chemical reagents were used as received. Elemental analyses for CHN were made by the Microanalytical Service of the Instituto Superior Técnico. The IR spectra were taken on a BIO-RAD FTS 3000MX (Bio-Rad Laboratories Inc., Hercules, CA, USA) instrument in KBr pellets.

### 2.1. Synthesis of [Cu_2_(HL^1^)(L^1^)(N_3_)_3_]∙2H_2_O 

The 1-(2-aminoethyl)piperazine (1 mmol, 0.13 mL) and salicylaldehyde (1 mmol, 0.11 mL) were dissolved in 15 mL of methanol. The resulting light-yellow solution was stirred at 50–60 °C for 30 min. Then, 0.17 g (1 mmol) of CuCl_2_·2H_2_O in 3 mL of methanol, 0.27 g (1 mmol) of FeCl_3_·6H_2_O in 3 mL of methanol, and 1.3 g (20 mmol) of NaN_3_ in 2 mL of water were added dropwise in this order. After the addition of the solutions of salts, the reaction mixture got green colour, which turned to dark-brown after the addition of sodium azide. The resulting dark-brown solution was stirred for 30 min, then filtered and kept at room temperature. The powder, which was found to be a mixture of brown and green microcrystals, was obtained in 1 day. Dark-green crystals suitable for single-crystal X-ray study were formed from the filtrate after 1 month. Yield: 0.16 g, 42% (based on copper chloride). Anal. calc. for Cu_2_C_26_H_40_N_15_O_4_ (M = 753.803): C, 41.43%; N, 27.87%; H, 5.35%. Found: C, 41.4%; N, 27.6%; H, 5.4%.

### 2.2. Synthesis of [Cu_2_L^2^(N_3_)_2_]∙H_2_O 

The 1-(2-aminoethyl)piperazine (1 mmol, 0.13 mL) and salicylaldehyde (1 mmol, 0.11 mL) were dissolved in 15 mL of DMF. The resulting light-yellow solution was stirred at 50–60 °C for 30 min. Then, 0.46 g (2 mmol) of Cu(NO_3_)_2_·2.5H_2_O in 5 mL of DMF and 1.3 g (20 mmol) of NaN_3_ in 5 mL of DMF/water mixture (1:1) were added dropwise in this order. The resulting dark-brown solution was stirred for 30 min, then filtered and kept at room temperature. Dark-green crystals suitable for single-crystal X-ray study were formed in 1 month. Yield: 0.12 g, 17% (based on copper nitrate). Anal. calc. for Cu_2_C_27_H_38_N_12_O_3_ (M = 705.78): C, 45.95%; N, 23.82%; H, 5.43%. Found: C, 45.1%; N, 23.3%; H, 5.4%.

### 2.3. Crystallography

The single-crystal X-ray data for **1** and **2** were acquired on a Bruker AXS KAPPA APEX II (Bruker AXS Inc., Madison, WI, USA) diffractometer. Cell parameters were retrieved and refined using the Bruker SAINT (Bruker AXS Inc., Madison, WI, USA) program. SADABS (Bruker AXS Inc., Madison, WI, USA) was used for correction of absorption [27]. Both structures were solved by direct methods and refined against *F*^2^ using the SHELX-2018/3 (University of Göttingen, Göttingen, Germany) program [28] (Table 1). The hydrogen atoms of water molecules (in **1** and **2**) and aminogroup of piperazine ligand (in **2**) were localized and refined (O−H and N–H distances were restrained to 0.85 and 0.91 Å, respectively). The interatomic H···H separations in water molecules were restrained to 1.38 Å. The remaining H-atoms were placed at calculated positions and refined using the riding model with *U*_iso_ = 1.2*U_eq_*. Hirshfeld analysis and surface visualisation were made using the CrystalExplorer (University of Western Australia, Crawley, Australia) 17.5 program [29]. 

### 2.4. DFT Calculations

Single-point and broken-symmetry [30,31,32] calculations were performed by using the B3LYP/G functional [33,34,35,36] with the TZVPP basis set for the copper atoms and coordination sphere, and SVP basis set for all other atoms. The ORCA 4.2.1 (Max Planck Institute for Coal Research, Mülheim an der Ruhr, Germany) package was used [37] with integration grids Grid4. The chain-of-spheres RIJCOXS approximation was applied, with the support of the auxiliary basis def2/J [38]. The X-ray atom coordinates of **1** and **2** were used without geometry optimization. The dummy H atoms (used for generation of structure fragments for H-bonded synthon in **2**) were generated by using the Avogadro 1.2.0 (University of Pittsburgh, Pittsburgh, PA, USA) program [39]. The exchange couplings were determined according to the formalism *J*_AB_ = −(*E*_HS_ − *E*_BS_) / (*S*_A_ + *S*_B_)^2^ (where *E*_HS_ and *E*_BS_ are energies of high-spin and broken-symmetry states, respectively) [40,41,42,43]. The isosurfaces of spin densities were drawn using the VESTA 3.5.2 (National Institute for Materials Science, Tsukuba, Japan) program [44]. Shortened examples of the ORCA inputs for single point and broken symmetry calculations are given in the Listings S1 and S2.

### 2.5. Catalytic Oxidation of Cyclohexane

An amount of 5 µmol of solid catalyst was weighed into a flask. Then, 4.4 mL of CH_3_CN, 50 µmol of promoter (pyridine or HNO_3_ in the form of stock solutions in acetonitrile), 0.5 mL of nitromethane solution (internal standard; 1 mL of CH_3_NO_2_ mixed with 9 mL of CH_3_CN), 108 µL (1 mmol) of cyclohexane, and 0.28 mL (5 mmol) of H_2_O_2_ (50% aqueous) were added in this order at 50 °C under stirring (CAUTION: the combination of air or molecular oxygen and H_2_O_2_ with organic compounds at elevated temperatures may be explosive!). Aliquots (ca. 0.5 mL) of reaction mixture were transferred, upon cooling, into a vial containing an excess (ca. 150 mg) of solid Ph_3_P (according to the method developed by Shul’pin [45]) and then analysed directly by gas chromatography (GC) or gas chromatography–mass spectrometry (GC–MS) techniques. The aliquots containing nonreduced peroxides may show incorrect amounts of cyclohexanol and cyclohexanone due to spontaneous decomposition of cyclohexyl hydroperoxide in a GC injector and/or column [45].

### 2.6. Catalytic Amidation 

The reactions were carried out under N_2_ atmosphere in a Schlenk tube under stirring and control of temperature. First, 12.5 µmol of the catalyst and 0.5 mmol of benzamide were weighted into the Schlenk tube in solid form. Then 1 mL of chlorobenzene and 0.54 mL (5 mmol) of cyclohexane were added in this order. Then 184 µL (1 mmol) of the oxidant *^t^*BuOO*^t^*Bu was added at room temperature. The mixture was frozen with liquid nitrogen. Then the Schlenk tube was pumped and filled with N_2_ a couple of times. The frozen mixture was left to warm up under vacuum, and the above procedure was repeated. After that, the Schlenk tube was filled with N_2_ and heated at 90 °C. After 24 h, the reaction mixtures were cooled to room temperature. Then 10 mL of acetonitrile and 100 µL of α,α,α-trifluorotoluene (used as a GC internal standard) were added. The resulting mixture was analysed by GC/GC–MS techniques.

### 2.7. Gas Chromatography

A PerkinElmer Clarus 500 (PerkinElmer, Waltham, MA, USA) gas chromatograph (SGE BP-20 capillary GC column (Trajan, Melbourne, Australia) 30 m × 0.22 mm × 25 μm dimensions) equipped with a FID detector and a PerkinElmer Clarus 600 (PerkinElmer, Waltham, MA, USA) gas chromatograph (two SGE BPX-5 capillary GC columns (Trajan, Melbourne, Australia), the same dimensions) equipped with a FID detector and with a PerkinElmer Clarus 600 C (PerkinElmer, Waltham, MA, USA) electron impact mass spectrometer were used for quantitative and qualitative analyses of the catalytic mixtures (helium carrier gas was used). All Electron Ionization (EI) mass spectra were recorded using 70 eV ionization energy. The identification of product peaks at the chromatograms was made on the basis of the NIST v. 2.2 mass spectral database (PerkinElmer TurboMass v. 5.4.2.1617 software was used).

## 3. Results

### 3.1. Synthesis and Spectroscopic Analysis

**Figure 1:**
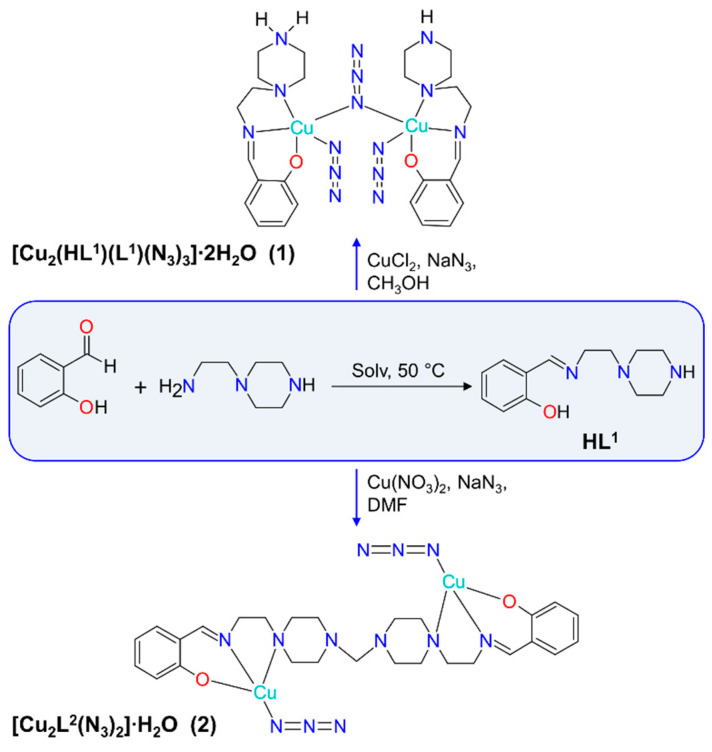
Schematic representation of the formation of **1** and **2**.

The complexes **1** and **2** were prepared employing a stepwise synthetic approach (Figure 1). The first step was the in situ formation of a Schiff base proligand by condensation of salicylaldehyde and 1-(2-aminoethyl)piperazine. In the next step, the obtained in situ Schiff base proligand was reacted with a metal precursor. Such method of complex preparation is commonly used in the synthesis of coordination compounds and allows the use of the Schiff base ligand immediately after its formation. For **1**, the interaction of copper(II) and iron(III) chlorides with NaN_3_ in a methanol solution of the Schiff base ligand using the molar ratio of CuCl_2_:FeCl_3_:Ligand = 1:1:1 resulted in a dark-brown solution. The powder (later it was found to be a mixture of brown and green microcrystals) precipitated in 1 day, while dark-green microcrystals of **1** were formed from the filtrate in 1 month. Complex **2** was formed by means of the interaction of copper(II) nitrate with NaN_3_ in a DMF solution of the Schiff base using the molar ratio of Cu(NO_3_)_2_:Ligand = 2:1. The dark-brown solution was heated and magnetically stirred for 30 min in open air, then filtered to remove undissolved solid and kept at room temperature until dark-green crystals of **2** suitable for X-ray crystallographic study were formed (ca. 1 month). Unexpectedly, single-crystal X-ray analysis of **2** disclosed a new organic ligand, (L^2^)^2−^, formed in situ (Figure 1). The formation of **2** can be understood by assuming a partial decomposition of DMF solvent into formaldehyde and dimethylamine with subsequent C–N coupling between formaldehyde and piperazine aminogroups [46]. This process is known for piperidine and piperazine chemistry [47,48,49]. The search via the Cambridge Structural Database (CSD, version 5.41, August 2020) [50,51] revealed six crystal structures of piperazine-based organic and coordination compounds [52], three of which were obtained by coupling piperazine groups in DMF media.

The IR spectra of **1** and **2** in the 4000–400 cm^−1^ (Figures S1 and S2) range indicate the presence of the Schiff base ligands. The broad bands of medium intensity around 3400 cm^−1^ were assigned to *ν*(O–H) vibrations of uncoordinated water molecules. The strong bands at 1638 (**1**) and 1630 cm^−1^ (**2**) were assigned to *ν*(C=N) stretching vibrations of the Schiff bases. The presence of both terminal and end-on bridging azide ligand in **1** was identified by the strong *ν*_as_(N_3_) absorption peaks at 2130 and 2053 cm^−1^, respectively [53]. The very strong *ν*_as_(N_3_) absorption peak at 2046 cm^−1^ showed the presence of the terminal azide ligand in **2**.

### 3.2. Crystal Structures

**Figure 2:**
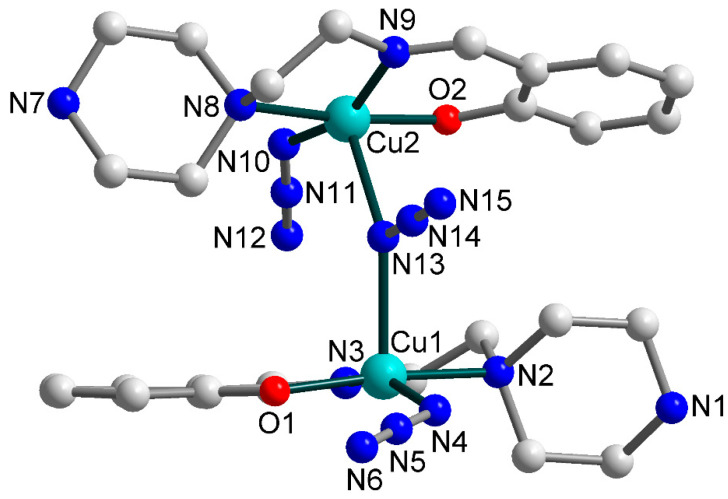
Molecular structure of **1** (building unit) with the atom numbering scheme. H atoms and uncoordinated water molecules are omitted for clarity. Colour scheme: Cu, cyan; O, red; N, blue; C, grey.

**Figure 3:**
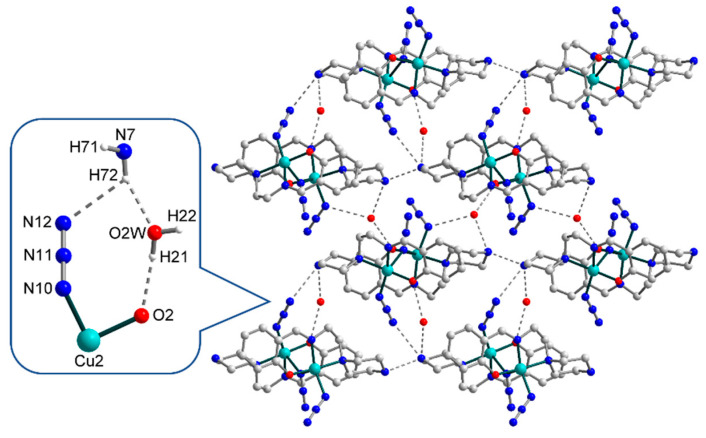
Representation of the supramolecular layer in **1**, viewed down the crystallographic *b* axis, with the enlarged fragment showing the supramolecular synthon formed by H-bonding between Schiff base ligands, terminal azide group, and uncoordinated water molecule. The hydrogen atoms are omitted for clarity. Colour scheme: Cu, cyan; O, red; N, blue; C, grey.

The crystal structure of [Cu_2_(HL^1^)(L^1^)(N_3_)_3_]∙2H_2_O (**1**) consists of dinuclear molecules, where copper(II) atoms are joined by end-on azide bridge (Figure 2), and two uncoordinated water molecules, which join into supramolecular two-dimensional layers assisted by strong hydrogen bonds (Figure 3). Although the hydroxyl groups of the Schiff bases deprotonate during the synthesis of **1**, one of two ligand molecules remains uncharged (HL^1^) because the secondary amine of the piperazine group is protonated. Compound **1** contains two crystallographically independent copper(II) atoms, Cu1 and Cu2. Each of them has a distorted square-pyramidal ON_4_ coordination environment (Figure 2) formed by the donor atoms from tridentate chelating Schiff base ligand occupying three of the equatorial metal coordination sites, while the remaining basal position is engaged with the N atom from terminal azide and the axial one with the N atom from bridging azide ligand. The equatorial Cu–X (X = O, N) bond lengths assume values in the range of 1.927(6)–2.101(6) Å, while the apical Cu–N ones are 2.329(7) and 2.390(7) Å, for Cu1 and Cu2, respectively (Table 2). The N/O–Cu–N_trans_ angles lie in the range from 157.7(4) to 174.0(3)°. The Cu∙∙∙Cu separation within the dinuclear molecule is 4.389(0) Å.

**Figure 4:**
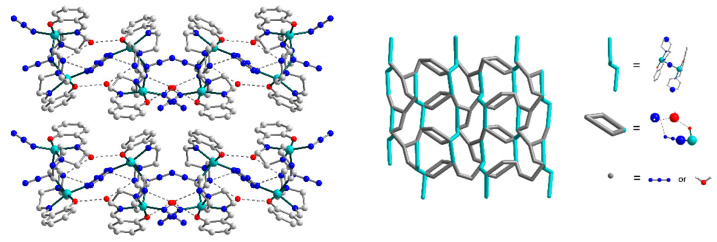
Packing of the supramolecular layers in **1** along the *a* axis. The hydrogen atoms are omitted for clarity. Colour scheme: Cu, cyan; O, red; N, blue; C, grey. Right: simplified topology of the layer in **1**.

The strong H-bonds of three types, O–H···O, N–H···O, and N–H···N [O2W–H21···O2, D–A 2.817(0) Å, D–H···A = 160.79(0)°; N7–H72···O2W, D–A 2.878(0) Å, D–H···A 152.63(0)°; N7–H72···N12, D–A 3.152(0) Å, D–H···A 121.71(0)°], involving oxygen and nitrogen atoms from the O2-phenolate and N7-amine moieties of Schiff base ligands, respectively, as well as N12 atom from a terminal azide ligand and O2W atom from an uncoordinated water molecule, form the eight-membered supramolecular synthon (Figure 3, in enlargement), which joins the neighbouring dinuclear molecules into supramolecular chains. Besides, these chains are strengthened by N–H···N [N7–H71···N1, D–A 2.713(0) Å, D–H···A 158.31(1)°] interactions between N7- and N1-amine moieties of Schiff bases. Moreover, the O1W atoms of other solvated water molecules link supramolecular chains into two-dimensional layers, showing the simultaneous formation of three H-bonds, namely, O–H···O, O–H···N, and N–H···O [O1W–H12···O1, D–A 2.769(0) Å, D–H···A = 159.48(0)°; O1W–H11···N4, D–A 3.187(0) Å, D–H···A 149.03(1)°; N1–H1···O1W, D–A 2.823(0) Å, D–H···A 134.67(0)°]. Further growth of the dimensionality of the supramolecular 2D complex is not observed due to steric limitations: the bulky Schiff base ligands prevent the formation of H-bonds between the layers (Figure 4, left). Rather, a complex topology of 2D layers in structure **1** can be visualized by simplifying the structure and replacing the ligands with dots (Figure 4, right).

**Figure 5:**
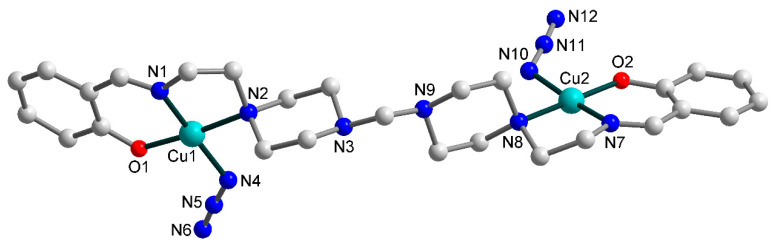
Molecular structure of **2** (building unit) showing the atom numbering. H atoms and uncoordinated water molecule are omitted for clarity. Colour scheme: Cu, cyan; O, red; N, blue; C, grey.

**Figure 6:**
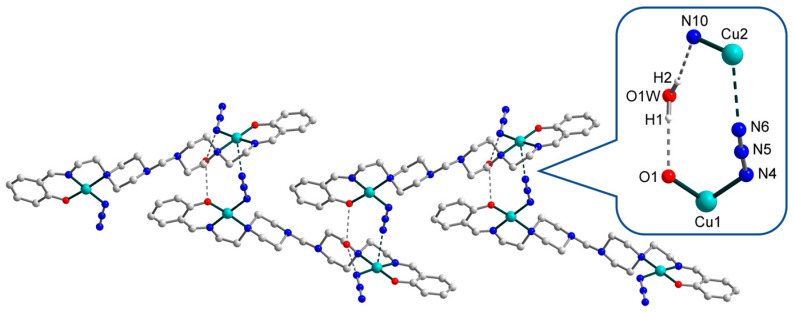
Representation of the supramolecular chain in **2**, viewed along the crystallographic *c* axis, with the inset showing synthon formed by H-bonding between Schiff base ligand, terminal azide group, and uncoordinated water molecule, as well as weak Cu–N contact. The hydrogen atoms are omitted for clarity. Colour scheme: Cu, cyan; O, red; N, blue; C, grey.

The X-ray analysis reveals that [Cu_2_L^2^(N_3_)_2_]∙H_2_O (**2**) is formed by a dinuclear molecule (Figure 5) and uncoordinated water, which form supramolecular chains due to hydrogen bonds (Figure 6). The Schiff base ligand, (L^2^)^2−^, in **2** is doubly deprotonated and has two coordination sites having the tridentate chelating (N,N,O) coordination mode (Figure 5). Thus, similar to **1**, the Schiff base predetermines the structure type formation and compensates the metal ion charge as well. Each of the two crystallographically independent copper(II) atoms, Cu1 and Cu2, has distorted square-planar geometry with an ON_3_ donor set formed by the N,O-donor atoms of the Schiff base and terminal azide ligands. The Cu–X (X = O, N) bond lengths in **2** range from 1.890(4) to 2.080(4) Å, while the O(N)–Cu–N_trans_ angles vary from 171.2(2) to 176.22(17)° (Table 3). 

**Figure 7:**
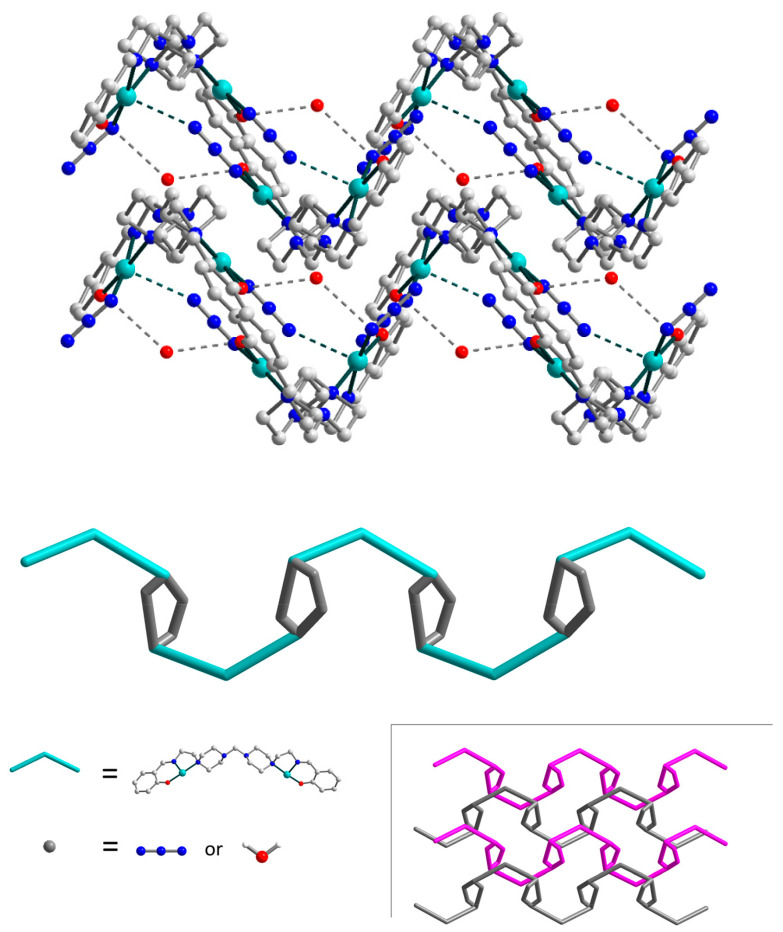
Top: packing of the supramolecular chains in **2** along the *b* axis. The hydrogen atoms are omitted for clarity. Bottom: simplified topology of **2**. Colour scheme: Cu, cyan; O, red; N, blue; C, grey.

The uncoordinated water molecules tie complex molecules of **2** together, forming 1D supramolecular chains by means of strong hydrogen bonding between the oxygen atoms of the Schiff bases and the nitrogen atoms of N_3_^−^ anions (Figure 6) [O1W–H1···O1, D–A 2.908(0) Å, D–H···A = 157.66(1)°; O1W–H2···N10, D–A 3.010(9) Å, D–H···A = 167.82(1)°]. Moreover, a weak contact of 2.940(7) Å exists between Cu2 atom and N6 atom from azide anion, which additionally reinforces polymeric chains and participates in the formation of the 10-membered supramolecular synthon (Figure 6, enlargement). The bond angles N6(3)···Cu2–X (X = N7, N8, N10, O2) in the range from 84.41(0)° to 97.85(0)° also confirm the existence of this contact. Thus, in fact, Cu2 atom has a distorted pyramidal (4+1) coordination environment. The intermolecular Cu···Cu separation is 11.209(2) Å. The supramolecular chains in **2** are densely packed, revealing an overall zigzag shape along the *b* axis (Figure 7). A simplified topology of 1D chains of **2** is shown in Figure 7, bottom.

### 3.3. Hirshfeld Surface Analysis

**Figure 8:**
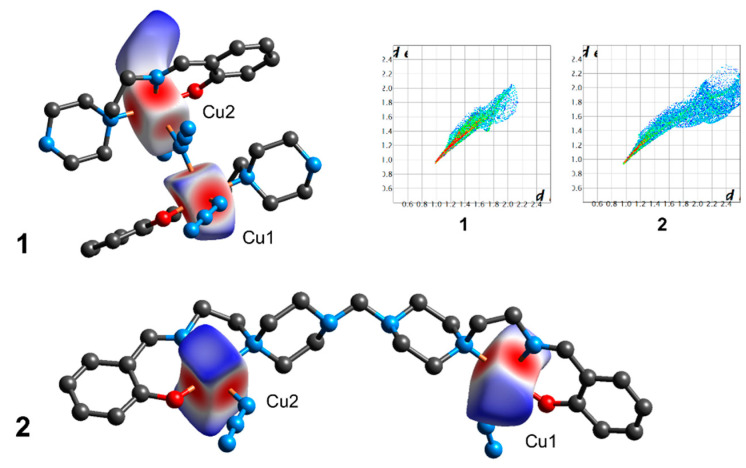
The Hirshfeld surface of copper centres in **1** and **2**. The coloured map corresponds to a normalized contact distance (*d*_norm_), ranging from −0.67 to 0.87 (for **1**) and −0.69 to 1.64 (for **2**). The inset shows the fingerprint plots (*d*_e_ vs. *d*_i_, Å) for each copper atom individually.

Analysis of the Hirshfeld surface (HS) [54] was performed to visualize the differences in coordination environments around crystallographically independent copper centres. The normalized contact difference (*d*_norm_) surfaces for **1** and **2** are shown in Figure 8. The shapes of Hirshfeld surfaces for all copper centres agree with their coordination environments (square-pyramidal ones, differing by apical distances). The surfaces of crystallographically independent copper centres in **1** reveal a significant difference in the apical positions (Figure 8). In contrast, the HS plots for both copper centres in **2** are similar. The fingerprint plots [54] for **1** and **2** are depicted in Figure 8, inset. The outer surface contacts are constructed mainly of those with N (65.1 and 44.5%), O (18.3 and 16.3%), H (14.7 and 38.5%), and C ones (1.9% and 0.7% for **1** and **2**, respectively). As can be seen, the contribution of Cu···H contacts is much higher for **2**, while structure **1** shows a larger amount of Cu···N contacts.

### 3.4. DFT Calculations

**Figure 9:**
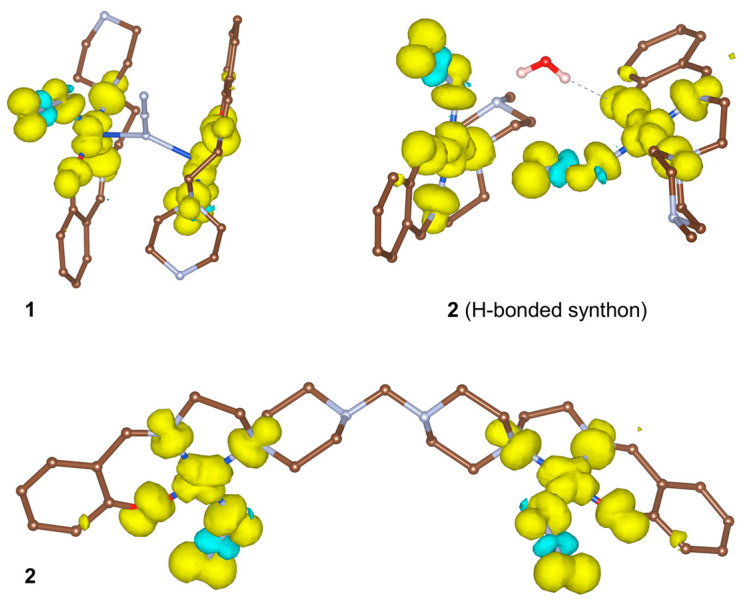
Isosurfaces of the DFT calculated spin densities for the triplet state of **1** and **2** (molecular dinuclear and H-bonded dimeric synthon) with a cutoff value of 0.002 e *a*_0_^3^ (yellow and blue correspond to a positive and negative density, respectively).

Single-point and broken-symmetry DFT calculations were used to evaluate the spin structures of **1** and **2**. In all cases, the highest Mulliken spin populations were located on copper centres as well as on coordinated N,O-atoms within the equatorial planes (Figure 9, Listings S3–S7). Surprisingly, the bridging azide group in **1** revealed nearly zero spin density on its nitrogen atoms (Listing S3), suggesting negligible magnetic exchange between the copper centres. The magnitude of singlet–triplet splitting in **1** was evaluated by broken-symmetry DFT calculations, which gave the *J*_CuCu_ value of −0.44 cm^−1^. The correctness of these calculations was confirmed by applying the same methodology towards the estimation of a singlet–triplet splitting and spin structures in literature complexes bearing a similar Cu–(N_3_)–Cu fragment. The complex [CuL^b^(N_3_)]_2_ (HL^b^ = (2-[1-(2-dimethylaminoethylimino)ethyl]phenol) reveals a Cu–N–Cu angle of 118.5°, being slightly lower that the respective angle in **1** (136.8°) [55]. The calculated spin density discloses the mutual arrangement of magnetic orbitals different from **1** (Figure 9). The predicted magnetic exchange between unpaired electrons on copper centres, *J*_CuCu_ = 0.84 cm^−1^ (Figure S3, Listing S6), is very close to the experimentally determined value of −1.97 cm^−1^. Another example is the complex [Cu_2_(N_3_)(L^c^)_2_](ClO_4_)_3_ bearing the cagelike ligand *m*-bis[bis(1-pyrazolyl)methyl]benzene (L^c^) [56]. In this case, the DFT single-point calculations disclosed a significant spin density localized on the azide bridging ligand (Figure S4, Listing S7). The broken-symmetry DFT calculations suggested a quite strong antiferromagnetic exchange of −427.6 cm^−1^ (Listing S7), this result being similar to that obtained earlier [56]. Although the magnitude of the exchange was overestimated (the experimentally determined *J*_CuCu_ was −223 cm^−1^), the calculations correctly predicted the sign and tendency of the exchange. These results are in conformity with those previously reported and point out the correctness of the methodology chosen.

DFT calculations predict the magnetic exchange between copper centres in **2** to be very weak: *J*_CuCu_ = −1.54 and −2.02 cm^−1^ for interactions within the molecule of **2** (*d*(Cu···Cu) = 11.21 Å) and within the dimeric H-bonded synthon (*d*(Cu···Cu) = 6.53 Å), respectively (Figure 9). Although examples of significant long-range exchange interactions at more than 6 Å distance are known [57], the ligand in **2** and the H-bonded network in the synthon are poor transmitters of superexchange interactions; thus the magnetic couplings in the structure of **2** should be negligible.

### 3.5. Catalytic Oxidation of Cyclohexane

**Figure 10:**
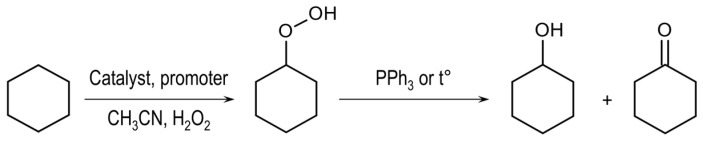
Catalytic oxidation of cyclohexane with H_2_O_2_, catalysed by complexes **1** and **2**.

The catalytic properties of **1** and **2** were investigated in the oxidation of cyclohexane (CyH) with H_2_O_2_ under mild conditions (atmospheric pressure and 50 °C temperature) in the presence of nitric acid or pyridine (Py) as promoters (Figure 10).

**Figure 11:**
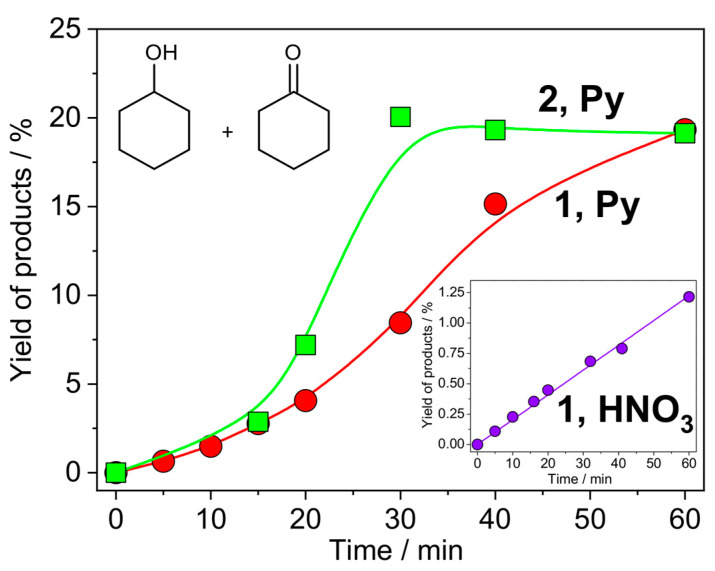
Accumulation of oxygenates (sum of cyclohexanol and cyclohexanone) over time in the oxidation of 0.2 M of cyclohexane with 1.0 M of H_2_O_2_ (50% aqueous) in the presence of promoter (1 × 10^−2^ M) catalysed by complex **1** or **2** (1 × 10^−3^ M) in acetonitrile at 50 °C.

Both complexes (0.14 mol% loading) reveal high activities in the oxidation of cyclohexane (CyH) with H_2_O_2_ (5 equiv.) in acetonitrile under mild conditions (50 °C temperature and atmospheric pressure) using pyridine (Py) as a promoting agent (5 mol%), reaching TONs (turnover numbers) up to 140. The highest reaction rate of 7.1 × 10^−5^ M·s^−1^ (supported by the yield of products of 21%) is observed at 30 min for the **2**/Py catalytic system (Figure 11). Pyridine is able to promote proton transfer steps that are required in the metal-catalysed formation of HO• from H_2_O_2_ [58,59]. This role can be further relevant because the N,N,O-ligands have their basic sites blocked by H-bonds and thus cannot effectively promote the above H^+^-transfer steps. Moreover, pyridine is able to coordinate metal centres, eventually favouring the formation of a catalytically active species [21,60,61,62,63]. 

Although nitric acid can act as an efficient promoting agent (e.g., for copper-catalysed oxidations with H_2_O_2_ [5,64]), in the present case, this promoter is much less efficient, leading to ca. 1% of yield and showing the two orders’ lower reaction rate of *W*_0_ = 6.8 × 10^−7^ M·s^−1^. This can be due to the preferable protonation of the azide ligands to the N,N,O-ones, which thus remain fully coordinated to the metal centre, without the formation of an unsaturated coordination environment.

**Figure 12:**
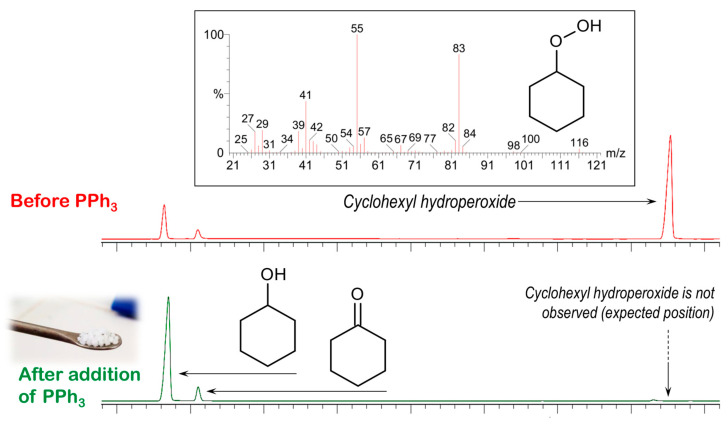
Fragment of the chromatogram of the reaction products in the oxidation of cyclohexane catalysed by **1** (conditions are as those stated in Figure 11 caption) at 1 h reaction time. The peaks of cyclohexanol and cyclohexanone, observed along with cyclohexyl hydroperoxide (top), represent the sums of the products contained in the aliquots and those due to partial decomposition of CyOOH in a hot GC injector and column.

The main reaction product is cyclohexyl hydroperoxide (CyOOH), as evidenced by the GC–MS analysis of the reaction mixtures [65,66,67], where the hydroperoxide was detected directly (Figure 12). The peak of Cy–OOH completely disappears after the addition of PPh_3_, which quantitatively reduces the hydroperoxide to the respective alcohol [45]. Observation of CyOOH as a major reaction product is expected for the reaction route where hydroxyl radical is a main C–H attacking species [5,14]. In this mechanism, a cyclohexane C–H bond is homolytically split to form the cyclohexyl radical Cy•. The latter reacts with dioxygen to form the peroxyl radical CyOO•, which could be reduced by a copper catalyst to produce the alkyl peroxyl anion CyOO^−^ and finally cyclohexyl hydroperoxide CyOOH. The reaction proceeds with a selectivity towards cyclohexanol and cyclohexanone of more than 95%. The by-product pattern, recorded after 24 h, reveals a complex mixture of cyclohexane diols, hydroxycyclohexanones, and other species (Figure S5). This pattern is expected for a hydroxyl radical attack of cyclohexane [20,66,68,69], in this way providing additional evidence for this type of oxidation mechanism.

### 3.6. Catalytic Amidation of Cyclohexane

**Figure 13:**
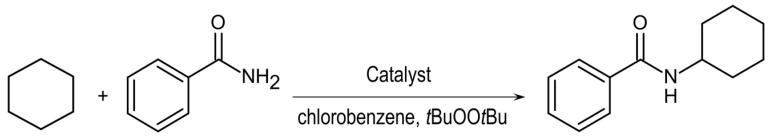
Catalytic amidation of cyclohexane, catalysed by **1**.

Complex **1**, revealing much higher solubility in acetonitrile and cyclohexane than **2**, was tested as a catalyst in the reaction of intermolecular amidation of cyclohexane in chlorobenzene medium (Figure 13). 

**Figure 14:**
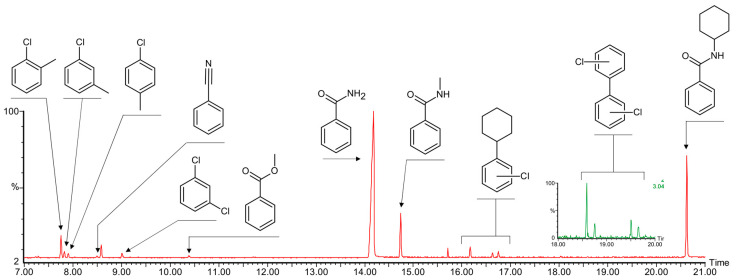
Fragment of the chromatogram showing the reaction products and by-products of the amidation of cyclohexane with benzamide catalysed by **1**. The initial parts of the chromatogram containing peaks of the solvent, substrate, and internal standard are omitted for clarity.

The reaction of benzamide with 10 equivalents of cyclohexane in the presence of 2 equivalents of oxidant (*^t^*BuOO*^t^*Bu, di-*tert*-butyl peroxide, DTBP) and catalyst **1** (2.5 mol% relative to benzamide) at 100 °C and under N_2_ atmosphere affords N-cyclohexyl benzamide (Figure 13). Chlorobenzene solvent was used due to its high boiling temperature and good solubility of the complex. The conversion of benzamide was 55% after 24 h, supported by a TON of 23. The reaction by-products are those formed through the methylation of chlorobenzene and benzamide via the attack of the methyl radical, typically forming in low quantities from the *t*BuOO*t*Bu oxidant during its thermal splitting [70]. The observation of chloro-2-cyclohexylbenzenes (Figure 14) suggests the participation of chlorobenzene radicals, appearing as a result of the reaction of *t*BuO• radical with chlorobenzene solvent. The search for dichloro-biphenyls disclosed traces of this by-product (Figure 14), thus confirming the participation of chlorobenzene radicals as intermediates.

**Figure 15:**
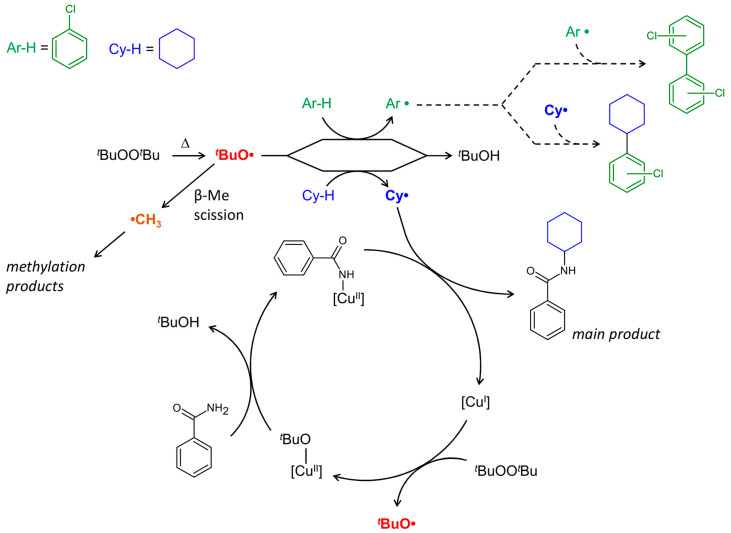
Proposed reaction mechanism for the cyclohexane amidation catalysed by **1** (schematically shown as [Cu]).

Based on previous studies [24,70] and considering the data obtained herein, a plausible reaction mechanism can be proposed (Figure 15), where the radical species and principal intermediates are shown. The reaction starts from the thermal splitting of DTBP, which becomes notable at temperatures higher than 90 °C. Hydrogen abstraction from cyclohexane by *t*BuO• radical affords cyclohexyl radical, which is trapped by a copper catalyst (Figure 15). Hence, the efficiency of the C–H amidation strongly depends on the affinity of a metal complex catalyst to alkyl radicals. The proposed mechanism foresees the change of copper oxidation state from Cu(II) to Cu(I) and vice versa (Figure 15). Such processes are expected for radical oxidative transformations catalysed by copper, where the change of the oxidation state occurs upon the reaction of a copper catalyst with peroxide and/or radical species [5,14,15]. Participation of both Cu(I) and Cu(II) intermediates in the radical amidation of cyclohexane was suggested earlier [70].

Recently, we reported polynuclear copper complexes bearing aminoalcohol ligands with bulky aliphatic substituents, catalysing cyclohexane amidation with 20% conversation of benzamide under similar conditions [24]. In the present work, catalyst **1** shows a considerably higher activity (55% conversion), at the same time affording higher amounts of methylation products (Figure 15). Elevated amounts of methylated products, particularly N-methylbenzamine, suggest that **1** acts as an efficient trap for both methyl and cyclohexyl radicals. 

## 4. Conclusions

We described the synthesis and crystal structures of two novel supramolecular compounds of copper, [Cu_2_(HL^1^)(L^1^)(N_3_)_3_]∙2H_2_O (**1**) and [Cu_2_L^2^(N_3_)_2_]∙H_2_O (**2**), which were synthesized by reacting the in situ prepared Schiff base proligands HL^1^ or H_2_L^2^ with copper salts in nonaqueous media in the presence of an azide source. For **2**, under the experimental conditions, in DMF medium, the piperazine groups of HL^1^ undergo coupling with the formation of the proligand H_2_L^2^. The crystal structures of the complexes were determined by X-ray diffraction. The crystal structure of **1** features dinuclear molecules joined by strong hydrogen bonds into 2D layers of complex topology. In contrast, dinuclear copper units in the structure of **2** self-organize into 1D polymeric chains. Despite the significant difference in the intermetallic distances, broken-symmetry DFT calculations disclosed surprisingly small singlet–triplet splitting in both **1** and **2**. Catalytic studies revealed that **1** and **2** act as efficient catalysts in the oxidation of cyclohexane with H_2_O_2_, promoted by pyridine, while nitric acid promoter was found to be much less efficient. Cyclohexyl hydroperoxide was directly detected by GC–MS technique, confirming a free radical catalytic mechanism. Complex **1**, possessing sufficient solubility in chlorobenzene, also shows a significant catalytic activity towards amidation of cyclohexane with benzamide, where a key role of the copper catalyst concerns trapping free cyclohexyl radicals, bringing them into reaction with benzamide.

## Figures and Tables

**Table 1:** Crystal data and structure refinement for **1** and **2**.

	1	2
Empirical Formula	C_26_H_41_Cu_2_N_15_O_4_	C_27_H_38_Cu_2_N_12_O_3_
Formula Weight	754.82	705.77
Crystal System	Orthorhombic	Monoclinic
Space Group	*P*bca	*P* 21/*c*
*a*/Å	13.6181(10)	11.3959(16)
*b*/Å	19.6836(14)	19.983(3)
*c*/Å	25.2572(18)	13.3403(17)
*α*/°	90	90
*β*/°	90	90.335(6)
*γ*/°	90	90
*V*/Å^3^	6770.3(8)	3037.8(7)
*Z*	8	4
Calculated Density/g cm^−3^	1.481	1.543
*T*, K	296(2)	296(2)
*μ*(Mo-K_α_)/mm^−1^	1.313	1.452
*F*(000)	3136	1464
Reflections Collected/Unique	44613/5951	21704/5812
*R*_int_	0.1175	0.1057
Reflections with *F*^2^ > 2*σ*(*F*^2^)	3586	2005
*Θ*_min_*, Θ*_max/_°	2.199, 25.014	3.220, 26.373
*R*_1_, *F*^2^ > 2*σ*(*F*^2^)	0.0743	0.0486
*wR*_2_ (all data)	0.2190	0.1192
*GoF*	1.096	0.729
Radiation	Mo Kα	Mo Kα
CCDC numbers	2036334	2036341

**Table 2:** Selected geometrical parameters (distances/Å and angles/°) for **1**.

Cu1–O1	1.940(6)	Cu2–O2	1.927(6)
Cu1–N2	2.101(6)	Cu2–N8	2.088(6)
Cu1–N3	1.967(6)	Cu2–N9	1.962(7)
Cu1–N4	1.996(7)	Cu2–N10	1.949(8)
Cu1–N13	2.329(7)	Cu2–N13	2.390(7)
O1–Cu1–N2	172.9(3)	O2–Cu2–N8	174.0(3)
O1–Cu1–N3	92.2(3)	O2–Cu2–N9	92.4(3)
O1–Cu1–N4	92.9(3)	O2–Cu2–N10	91.8(3)
O1–Cu1–N13	94.0(2)	O2–Cu2–N13	92.9(2)
N2–Cu1–N3	82.9(3)	N8–Cu2–N9	83.8(3)
N2–Cu1–N4	90.0(3)	N8–Cu2–N10	90.0(3)
N2–Cu1–N13	91.9(3)	N8–Cu2–N13	91.6(2)
N3–Cu1–N4	158.9(3)	N9–Cu2–N10	157.7(4)
N3–Cu1–N13	101.2(3)	N9–Cu2–N13	88.8(3)
N4–Cu1–N13	98.9(3)	N10–Cu2–N13	112.8(4)

**Table 3:** Selected geometrical parameters (distances/Å and angles/°) for **2**.

Cu1–O1	1.905(4)	Cu2–O2	1.890(4)
Cu1–N1	1.924(5)	Cu2–N7	1.943(5)
Cu1–N2	2.080(4)	Cu2–N8	2.077(4)
Cu1–N4	1.949(4)	Cu2–N10	1.953(5)
O1–Cu1–N1	92.36(19)	O2–Cu2–N7	92.4(2)
O1–Cu1–N2	175.51(18)	O2–Cu2–N8	176.22(17)
O1–Cu1–N4	92.32(18)	O2–Cu2–N10	90.9(2)
N1–Cu1–N2	84.24(19)	N7–Cu2–N8	84.53(19)
N1–Cu1–N4	173.6(2)	N7–Cu2–N10	171.2(2)
N2–Cu1–N4	91.30(18)	N8–Cu2–N10	91.90(19)

## Supplementary Materials

The following are available online at https://www.mdpi.com/1996-1944/13/23/5435/s1, Figure S1: IR spectrum of 1, Figure S2: IR spectrum of **1**, Figure S3: Left: isosurface of the DFT calculated spin density for the triplet state of [CuL^b^(N_3_)]_2_ (CSD refcode RUYQAE) with the cutoff value of 0.002 e a_0_^3^ (yellow and blue correspond to a positive and negative density, respectively). Right: the same fragment, showing the atoms numbering scheme, Figure S4: Left: isosurface of the DFT calculated spin density for the triplet state of cation of [Cu_2_(N_3_)(L^c^)_2_](ClO_4_)_3_ (CSD refcode JUDNED) with the cutoff value of 0.002 e a_0_^3^ (yellow and blue correspond to a positive and negative density, respectively). Right: the same fragment, showing the atoms numbering scheme, Figure S5: Fragment of the chromatogram showing the by-products in the course of oxidation of cyclohexane (0.2 M) with H_2_O_2_ (1.0 M, 50% aqueous), in the presence of pyridine (1 × 10^−2^ M) catalysed by complex **1** (1 × 10^−3^ M) in acetonitrile (total volume of the reaction solution was 5 mL) at 50 °C, after 24 h, Listing S1: Shortened example of the ORCA input for DFT single point calculations of the triplet spin state of **1** (only metal atoms are shown; the inputs for **2** and literature complexes are similar), Listing S2: Shortened example of the ORCA input for DFT broken symmetry calculations of **1** (only metal atoms are shown; the inputs for **2** and literature complexes are similar). Listing S3: Selected output of the ORCA DFT broken symmetry calculations of **1**. The numberings of the atoms, corresponding to X-ray structures, are shows in red colour (for triplet state). Listing S4: Selected output of the ORCA DFT broken symmetry calculations of **2** (dimer). The numberings of the atoms, corresponding to X-ray structures, are shows in red colour (for triplet state), Listing S5: Selected output of the ORCA DFT broken symmetry calculations of **2** (H-bonded synthon, Figure 5). The numberings of the atoms, corresponding to X-ray structures, are shows in red colour (for triplet state), Listing S6: Selected output of the ORCA DFT broken symmetry calculations of [CuL^b^(N_3_)]_2_ (refcode RUYQAE, Figure S3). The numberings of the atoms, corresponding to X-ray structures, are shows in red colour (for triplet state). Listing S7: Selected output of the ORCA DFT broken symmetry calculations of cation of [Cu_2_(N_3_)(L^c^)_2_](ClO_4_)_3_ (refcode JUDNED, Figure S4). The numberings of the atoms, corresponding to X-ray structures, are shows in red colour (for triplet state).
