# Supplementary material for: Novel H-Bonded Synthons in Copper Supramolecular Frameworks with Aminoethylpiperazine-Based Ligands. Synthesis, Structure and Catalytic Activity
Source: Materials (Basel). 2020 Nov 29;13(23):5435. doi: 10.3390/ma13235435 (PMC7731324; doi:10.3390/ma13235435)
Supplement: Supplementary file 1 [file materials-13-05435-s001.pdf]

Novel H-bonded Synthons in Copper Supramolecular Frameworks with Aminoethylpiperazine-based Ligands. Synthesis, Structure and Catalytic Activity

Oksana V. Nesterova [1], Armando J.L. Pombeiro [1] and Dmytro S. Nesterov [1,2,*]

[1] Centro de Química Estrutural, Instituto Superior Técnico, Universidade de Lisboa, Av. Rovisco Pais, 1049-001 Lisboa, Portugal; oksana.nesterova@tecnico.ulisboa.pt (O.V.N.); pombeiro@tecnico.ulisboa.pt (A.J.L.P.)

[2] Peoples' Friendship University of Russia (RUDN University), 6 Miklukho-Maklaya st., Moscow 117198, Russia

* Correspondence: dmytro.nesterov@tecnico.ulisboa.pt (D.S.N.)

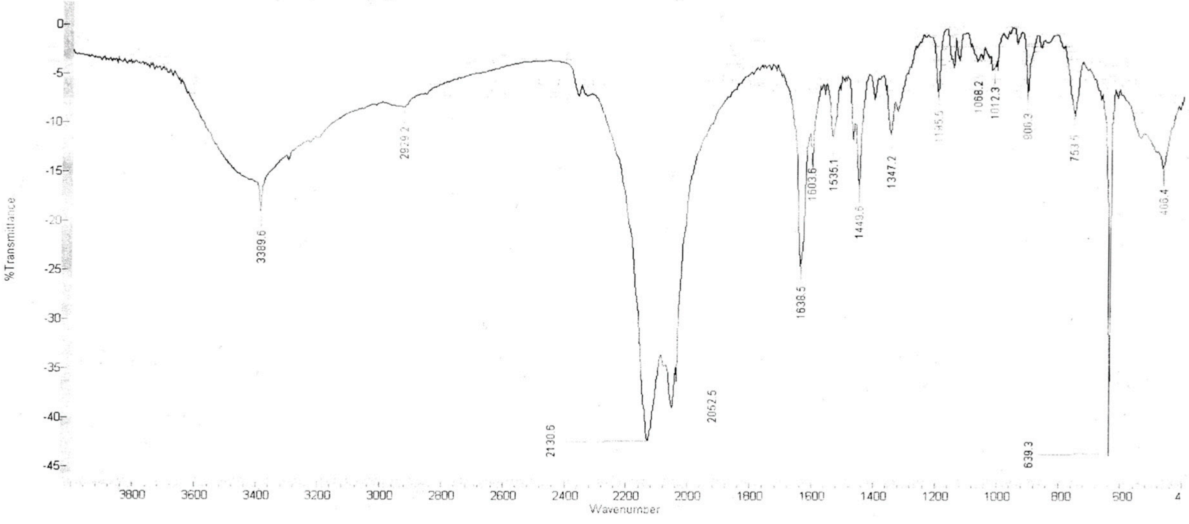

Figure S1. IR spectrum of **1**.

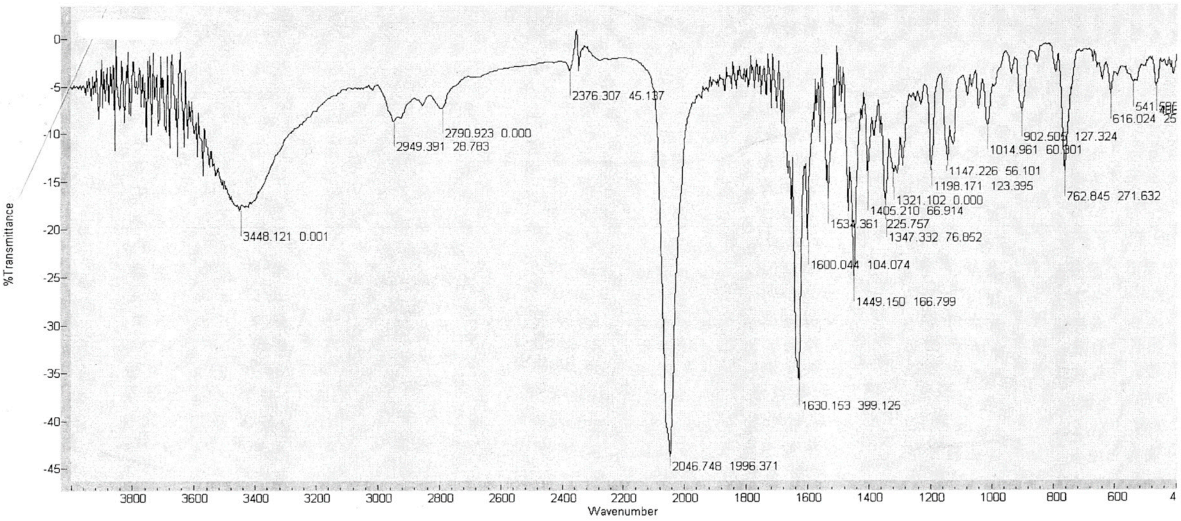

Figure S2. IR spectrum of **2**.

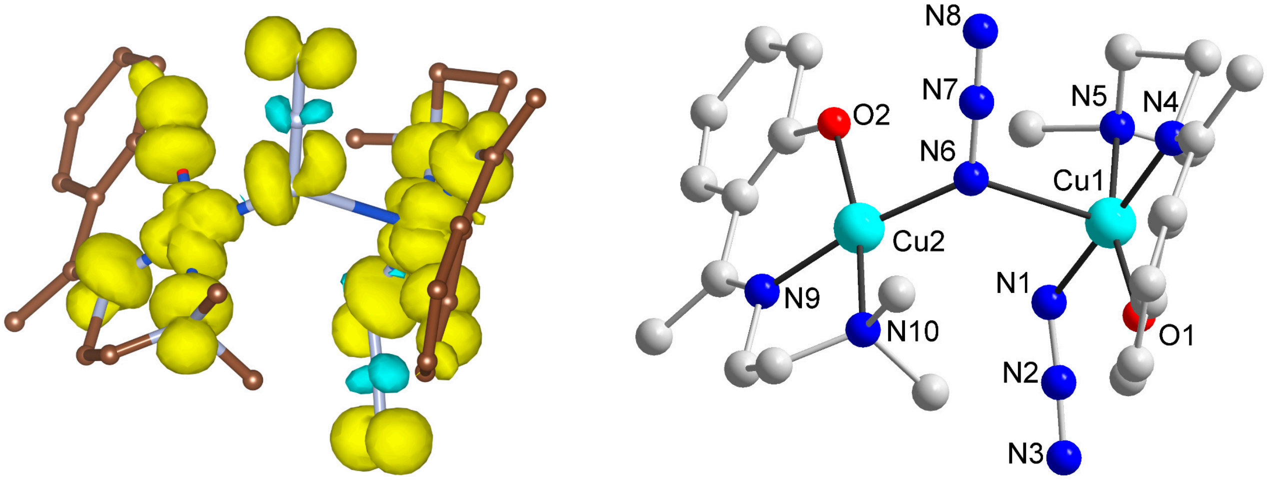

Figure S3. Left: isosurface of the DFT calculated spin density for the triplet state of $[CuL^b(N_3)]_2$ (CSD refcode RUYQAE) with the cutoff value of 0.002 e a_0^3 (yellow and blue correspond to a positive and negative density, respectively). Right: the same fragment, showing the atoms numbering scheme.

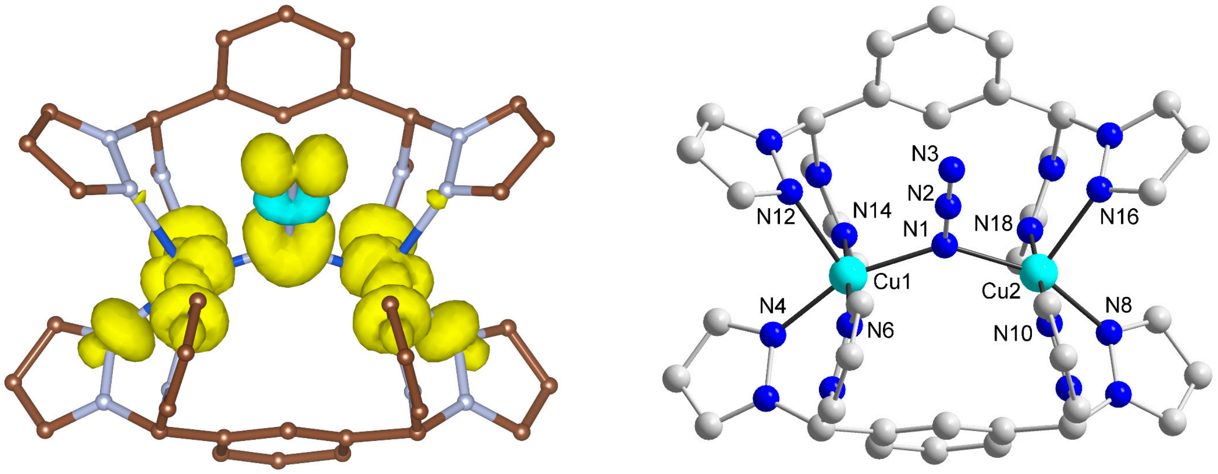

Figure S4. Left: isosurface of the DFT calculated spin density for the triplet state of cation of $[Cu_2(N_3)(L^c)_2](ClO_4)_3$ (CSD refcode JUDNED) with the cutoff value of 0.002 e a_0^3 (yellow and blue correspond to a positive and negative density, respectively). Right: the same fragment, showing the atoms numbering scheme.

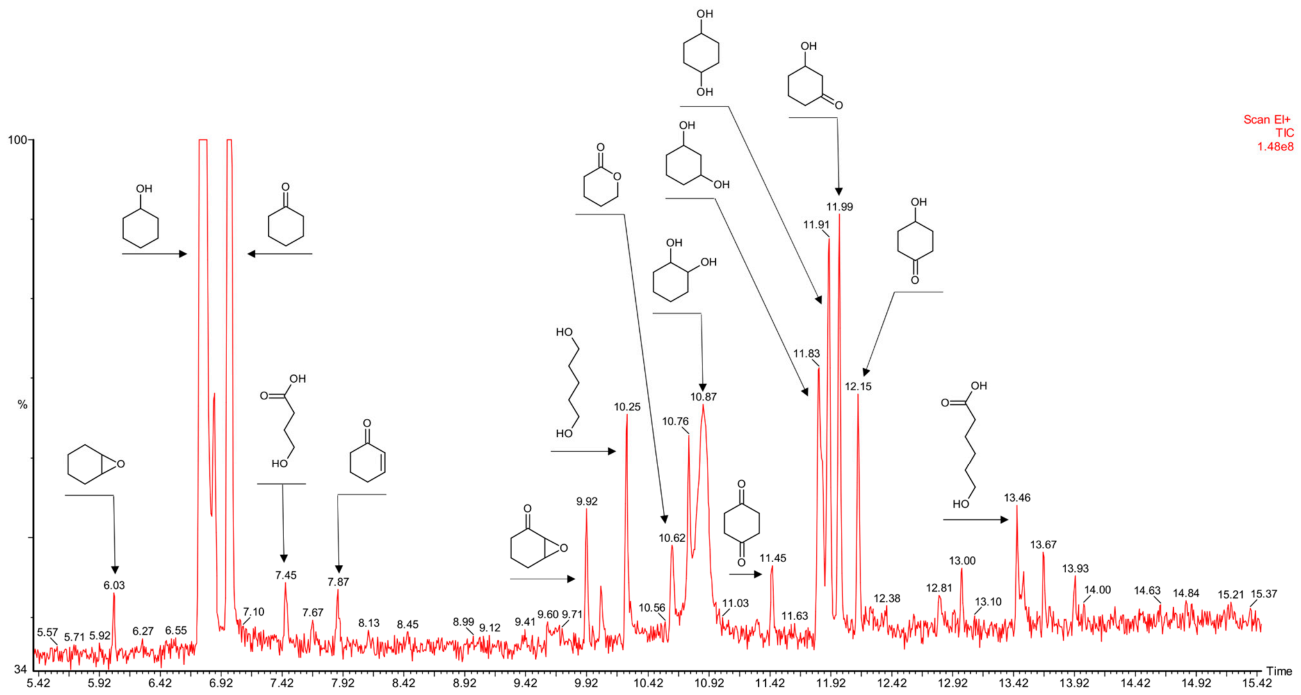

Figure S5. Fragment of the chromatogram showing the by-products in the course of oxidation of cyclohexane (0.2 M) with H_2O_2 (1.0 M, 50% aqueous), in the presence of pyridine (1×10^{-2} M) catalysed by complex **1** (1×10^{-3} M) in acetonitrile (total volume of the reaction solution was 5 mL) at 50 °C, after 24 h.

Listing S1. Shortened example of the ORCA input for DFT single point calculations of the triplet spin state of **1** (only metal atoms are shown; the inputs for **2** and literature complexes are similar).

```
! UKS B3LYP/G SVP TightSCF Grid4 NoFinalGrid rijcosx
! KeepDens
%plots
dim1 100
dim2 100
dim3 100
Format Gaussian_Cube
SpinDens("spindensity");
ElDens("electrondensity");
end
* xyz 0 3
Cu    4.97728   17.17650    8.84103 newgto "TZVPP" end
Cu    6.97478   13.41083    9.88516 newgto "TZVPP" end
```

Listing S2. Shortened example of the ORCA input for DFT broken symmetry calculations of **1** (only metal atoms are shown; the inputs for **2** and literature complexes are similar).

```
! UKS B3LYP/G SVP TightSCF Grid4 NoFinalGrid rijcosx
! KeepDens
%scf BrokenSym 1,1
end
%plots
dim1 100
dim2 100
dim3 100
Format Gaussian_Cube
SpinDens("spindensity");
ElDens("electrondensity");
end
* xyz 0 3
Cu    4.97728   17.17650    8.84103 newgto "TZVPP" end
Cu    6.97478   13.41083    9.88516 newgto "TZVPP" end
```

Listing S3. Selected output of the ORCA DFT broken symmetry calculations of **1**. The numberings of the atoms, corresponding to X-ray structures, are shows in red colour (for triplet state).

Triplet state:

```
----------------
TOTAL SCF ENERGY
----------------

Total Energy       :        -5264.67245769 Eh         -143259.02072 eV

Components:
Nuclear Repulsion  :         8186.43472829 Eh          222764.21407 eV
Electronic Energy  :       -13451.10718598 Eh         -366023.23479 eV
One Electron Energy:       -23592.09925228 Eh         -641973.65796 eV
Two Electron Energy:        10140.99206630 Eh          275950.42317 eV

Virial components:
Potential Energy   :       -10517.44423422 Eh         -286194.20745 eV
Kinetic Energy     :         5252.77177653 Eh          142935.18672 eV
Virial Ratio       :            2.00226560
```

```
DFT components:
N(Alpha)            :      186.999574214659 electrons
N(Beta)             :      184.999566132451 electrons
N(Total)            :      371.999140347110 electrons
E(X)                :     -315.703982678173 Eh
E(C)                :      -17.368282394442 Eh
E(XC)               :     -333.072265072616 Eh
DFET-embed. en.     :        0.000000000000 Eh

--------------------------------------------
MULLIKEN ATOMIC CHARGES AND SPIN POPULATIONS
--------------------------------------------
   0 Cu:     0.431282     0.564600     Cu1
   1 Cu:     0.475357     0.587620     Cu2
   2 O :    -0.460825     0.092633     O1
   3 O :    -0.479453     0.137242     O2
   4 N :    -1.011511     0.099262     N2
   5 N :    -0.436666     0.081394     N3
   6 N :    -0.410790     0.065952     N4
   7 N :     0.343289    -0.026267     N5
   8 N :    -0.450216     0.115561     N6
   9 N :    -0.962066     0.062652     N8
  10 N :    -0.448130     0.090515     N9
  11 N :    -0.457901     0.075346     N12
  12 N :    -0.598621     0.005050     N13
  13 N :    -0.534651     0.051292     N10
  14 N :     0.421354    -0.015356     N11
  15 N :     0.419475    -0.002493     N14
  16 N :    -0.445829    -0.002084     N15
```

Singlet state:

```
----------------
TOTAL SCF ENERGY
----------------

Total Energy        :        -5264.67245669 Eh         -143259.02069 eV

Components:
Nuclear Repulsion   :         8186.43472829 Eh          222764.21407 eV
Electronic Energy   :       -13451.10718498 Eh         -366023.23476 eV
One Electron Energy:        -23592.09937970 Eh         -641973.66143 eV
Two Electron Energy:         10140.99219472 Eh          275950.42667 eV

Virial components:
Potential Energy    :       -10517.44421683 Eh         -286194.20697 eV
Kinetic Energy      :         5252.77176014 Eh          142935.18628 eV
Virial Ratio        :            2.00226560


DFT components:
N(Alpha)            :      185.999567067696 electrons
N(Beta)             :      185.999573277139 electrons
N(Total)            :      371.999140344835 electrons
E(X)                :     -315.703982063720 Eh
E(C)                :      -17.368282686272 Eh
E(XC)               :     -333.072264749992 Eh
DFET-embed. en.     :        0.000000000000 Eh
```

```
-------------------------------------------
MULLIKEN ATOMIC CHARGES AND SPIN POPULATIONS
-------------------------------------------
   0 Cu:    0.431286    0.564557
   1 Cu:    0.475367   -0.587625
   2 O :   -0.460827    0.092648
   3 O :   -0.479462   -0.137251
   4 N :   -1.011515    0.099269
   5 N :   -0.436661    0.081217
   6 N :   -0.410796    0.065915
   7 N :    0.343316   -0.026248
   8 N :   -0.450222    0.115573
   9 N :   -0.962067   -0.062625
  10 N :   -0.448125   -0.090570
  11 N :   -0.457902   -0.075575
  12 N :   -0.598627    0.000029
  13 N :   -0.534652   -0.051143
  14 N :    0.421376    0.015516
  15 N :    0.419464   -0.000201
  16 N :   -0.445861   -0.001222

-----------------------------------------
BROKEN SYMMETRY MAGNETIC COUPLING ANALYSIS
-----------------------------------------

S(High-Spin)       =   1.0
<S**2>(High-Spin) =   2.0093
<S**2>(BrokenSym) =   1.0092
E(High-Spin)       = -5264.672458 Eh
E(BrokenSym)       = -5264.672457 Eh
E(High-Spin)-E(BrokenSym)= -0.0000 eV     -0.219 cm**-1 (FERROMAGNETIC coupling)

          --------------------------------------------------------
          | Spin-Hamiltonian Analysis based on H(HDvV)= -2J*SA*SB |
    -------                                                        -----------
    | J(1) =       0.22 cm**-1    (from -(E[HS]-E[BS])/Smax**2)                |
    | J(2) =       0.11 cm**-1    (from -(E[HS]-E[BS])/(Smax*(Smax+1))         |
    | J(3) =       0.22 cm**-1    (from -(E[HS]-E[BS])/(<S**2>HS-<S**2>BS))   |
    --------------------------------------------------------------------------

  J(1): (a) A.P. Ginsberg J. Am. Chem. Soc. 102 (1980), 111
        (b) L. Noodleman J. Chem. Phys. 74 (1981), 5737
        (c) L. Noodleman E.R. Davidson Chem. Phys. 109 (1986), 131
  J(2)  (d) A. Bencini D. Gatteschi J. Am. Chem. Soc. 108 (1980), 5763
  J(3)  (e) K. Yamaguchi Y. Takahara T. Fueno in: V.H. Smith (Ed.)
            Applied Quantum Chemistry. Reidel, Dordrecht (1986), pp 155
        (f) T.Soda et al. Chem. Phys. Lett., 319, (2000), 223
```

Listing S4. Selected output of the ORCA DFT broken symmetry calculations of **2** (dimer). The numberings of the atoms, corresponding to X-ray structures, are shows in red colour (for triplet state).

<u>Triplet state:</u>

```
----------------
TOTAL SCF ENERGY
----------------

Total Energy       :        -5137.90380775 Eh         -139809.47038 eV
```

```
Components:
Nuclear Repulsion  :         6261.88793635 Eh          170394.63344 eV
Electronic Energy  :       -11399.79174409 Eh         -310204.10383 eV
One Electron Energy:       -19566.17370479 Eh         -532422.65435 eV
Two Electron Energy:         8166.38196070 Eh          222218.55052 eV

Virial components:
Potential Energy   :       -10264.97025129 Eh         -279324.04110 eV
Kinetic Energy     :         5127.06644354 Eh          139514.57071 eV
Virial Ratio       :            2.00211376


DFT components:
N(Alpha)           :      178.999594289498 electrons
N(Beta)            :      176.999592385308 electrons
N(Total)           :      355.999186674806 electrons
E(X)               :     -303.918074842768 Eh
E(C)               :      -16.643278208130 Eh
E(XC)              :     -320.561353050898 Eh
DFET-embed. en.    :        0.000000000000 Eh

--------------------------------------------
MULLIKEN ATOMIC CHARGES AND SPIN POPULATIONS
--------------------------------------------
   0 Cu:    0.494473    0.517487    Cu2
   1 Cu:    0.487558    0.520039    Cu1
   2 O :   -0.429808    0.119540    O1
   3 O :   -0.461328    0.122877    O2
   4 N :   -0.491655    0.089225    N1
   5 N :   -1.007620    0.085631    N2
   6 N :    0.345386   -0.030626    N5
   7 N :   -0.439490    0.077757    N4
   8 N :   -0.387208    0.125609    N6
   9 N :   -0.993962    0.082059    N8
  10 N :   -0.469072    0.083096    N7
  11 N :    0.442066   -0.029167    N11
  12 N :   -0.535429    0.090224    N10
  13 N :   -0.382088    0.124341    N12
```

Singlet state:

```
----------------
TOTAL SCF ENERGY
----------------

Total Energy       :        -5137.90380425 Eh         -139809.47029 eV

Components:
Nuclear Repulsion  :         6261.88793635 Eh          170394.63344 eV
Electronic Energy  :       -11399.79174059 Eh         -310204.10373 eV
One Electron Energy:       -19566.17395308 Eh         -532422.66111 eV
Two Electron Energy:         8166.38221249 Eh          222218.55737 eV

Virial components:
Potential Energy   :       -10264.97025675 Eh         -279324.04125 eV
Kinetic Energy     :         5127.06645250 Eh          139514.57096 eV
Virial Ratio       :            2.00211375


DFT components:
N(Alpha)           :      177.999595446014 electrons
```

```
N(Beta)            :      177.999591228947 electrons
N(Total)           :      355.999186674961 electrons
E(X)               :     -303.918076520233 Eh
E(C)               :      -16.643278451636 Eh
E(XC)              :     -320.561354971869 Eh
DFET-embed. en.    :        0.000000000000 Eh

--------------------------------------------
MULLIKEN ATOMIC CHARGES AND SPIN POPULATIONS
--------------------------------------------
   0 Cu:    0.494468    0.517469
   1 Cu:    0.487554   -0.520025
   2 O :   -0.429808   -0.119542
   3 O :   -0.461327    0.122880
   4 N :   -0.491658   -0.089225
   5 N :   -1.007621   -0.085620
   6 N :    0.345381    0.030616
   7 N :   -0.439492   -0.077749
   8 N :   -0.387197   -0.125612
   9 N :   -0.993960    0.082053
  10 N :   -0.469076    0.083101
  11 N :    0.442063   -0.029153
  12 N :   -0.535427    0.090209
  13 N :   -0.382081    0.124348

------------------------------------------
BROKEN SYMMETRY MAGNETIC COUPLING ANALYSIS
------------------------------------------

S(High-Spin)       =   1.0
<S**2>(High-Spin) =   2.0108
<S**2>(BrokenSym) =   1.0108
E(High-Spin)       = -5137.903808 Eh
E(BrokenSym)       = -5137.903804 Eh
E(High-Spin)-E(BrokenSym)= -0.0001 eV     -0.769 cm**-1 (FERROMAGNETIC coupling)

          --------------------------------------------------------
          | Spin-Hamiltonian Analysis based on H(HDvV)= -2J*SA*SB |
    -------                                                    -----------
    | J(1) =       0.77 cm**-1    (from -(E[HS]-E[BS])/Smax**2)             |
    | J(2) =       0.38 cm**-1    (from -(E[HS]-E[BS])/(Smax*(Smax+1))      |
    | J(3) =       0.77 cm**-1    (from -(E[HS]-E[BS])/(<S**2>HS-<S**2>BS)) |
    -------------------------------------------------------------------------
```

Listing S5. Selected output of the ORCA DFT broken symmetry calculations of **2** (H-bonded synthon, Figure 6). The numberings of the atoms, corresponding to X-ray structures, are shows in red colour (for triplet state).

<u>Triplet state:</u>

```
----------------
TOTAL SCF ENERGY
----------------

Total Energy       :        -5254.86017186 Eh         -142992.01485 eV

Components:
Nuclear Repulsion  :         7454.40971009 Eh          202844.80064 eV
Electronic Energy  :       -12709.26988195 Eh         -345836.81549 eV
```

```
One Electron Energy:       -22112.76812792 Eh         -601719.01156 eV
Two Electron Energy:         9403.49824597 Eh          255882.19607 eV

Virial components:
Potential Energy   :       -10498.19411055 Eh         -285670.38495 eV
Kinetic Energy     :         5243.33393869 Eh          142678.37010 eV
Virial Ratio       :            2.00219826


DFT components:
N(Alpha)           :      189.000026529662 electrons
N(Beta)            :      187.000023300429 electrons
N(Total)           :      376.000049830091 electrons
E(X)               :     -316.274182536038 Eh
E(C)               :      -17.484070700688 Eh
E(XC)              :     -333.758253236726 Eh
DFET-embed. en.    :        0.000000000000 Eh

--------------------------------------------
MULLIKEN ATOMIC CHARGES AND SPIN POPULATIONS
--------------------------------------------
   0 Cu:    0.505424    0.530425    Cu1
   1 Cu:    0.482382    0.540425    Cu2
   2 O :   -0.484144    0.112264    O1
   3 O :   -0.453565    0.118658    O2
   4 O :   -0.081607    0.000092    O1W
   5 N :   -0.450395    0.084877    N7
   6 N :   -0.484054    0.094650    N1
   7 N :   -1.010668    0.092514    N2
   8 N :   -0.966491    0.082925    N8
   9 N :    0.371139   -0.023530    N5
  10 N :   -0.519580    0.079081    N10
  11 N :   -0.428843    0.064775    N4
  12 N :   -0.409440    0.114707    N6
  13 N :    0.411416   -0.026130    N11
  14 N :   -0.382523    0.112780    N12
```

Singlet state:

```
----------------
TOTAL SCF ENERGY
----------------

Total Energy       :        -5254.86016726 Eh         -142992.01472 eV

Components:
Nuclear Repulsion  :         7454.40971009 Eh          202844.80064 eV
Electronic Energy  :       -12709.26987735 Eh         -345836.81537 eV
One Electron Energy:       -22112.76844227 Eh         -601719.02012 eV
Two Electron Energy:         9403.49856493 Eh          255882.20475 eV

Virial components:
Potential Energy   :       -10498.19412653 Eh         -285670.38538 eV
Kinetic Energy     :         5243.33395927 Eh          142678.37066 eV
Virial Ratio       :            2.00219826


DFT components:
N(Alpha)           :      188.000023985539 electrons
N(Beta)            :      188.000025851525 electrons
N(Total)           :      376.000049837065 electrons
```

```
E(X)                :     -316.274186289118 Eh
E(C)                :      -17.484070707825 Eh
E(XC)               :     -333.758256996942 Eh
DFET-embed. en.     :        0.000000000000 Eh

--------------------------------------------
MULLIKEN ATOMIC CHARGES AND SPIN POPULATIONS
--------------------------------------------
   0 Cu:    0.505419    0.530421
   1 Cu:    0.482369   -0.540900
   2 O :   -0.484143    0.112270
   3 O :   -0.453564   -0.118622
   4 O :   -0.081607    0.000140
   5 N :   -0.450402   -0.084874
   6 N :   -0.484056    0.094650
   7 N :   -1.010663    0.092505
   8 N :   -0.966489   -0.083028
   9 N :    0.371121   -0.023877
  10 N :   -0.519561   -0.079057
  11 N :   -0.428845    0.065284
  12 N :   -0.409407    0.115199
  13 N :    0.411393    0.026113
  14 N :   -0.382513   -0.112764

------------------------------------------
BROKEN SYMMETRY MAGNETIC COUPLING ANALYSIS
------------------------------------------

S(High-Spin)       =   1.0
<S**2>(High-Spin) =   2.0101
<S**2>(BrokenSym) =   1.0101
E(High-Spin)       = -5254.860172 Eh
E(BrokenSym)       = -5254.860167 Eh
E(High-Spin)-E(BrokenSym)= -0.0001 eV     -1.010 cm**-1 (FERROMAGNETIC coupling)

          ---------------------------------------------------------
          | Spin-Hamiltonian Analysis based on H(HDvV)= -2J*SA*SB |
    -------                                                  -----------
    | J(1) =       1.01 cm**-1    (from -(E[HS]-E[BS])/Smax**2)             |
    | J(2) =       0.50 cm**-1    (from -(E[HS]-E[BS])/(Smax*(Smax+1))      |
    | J(3) =       1.01 cm**-1    (from -(E[HS]-E[BS])/(<S**2>HS-<S**2>BS)) |
    -------------------------------------------------------------------------
```

Listing S6. Selected output of the ORCA DFT broken symmetry calculations of $[CuL^b(N_3)]_2$ (refcode RUYQAE, Figure S3). The numberings of the atoms, corresponding to X-ray structures, are shows in red colour (for triplet state).

Triplet state:

```
----------------
TOTAL SCF ENERGY
----------------

Total Energy       :        -4913.00149433 Eh         -133689.56731 eV

Components:
Nuclear Repulsion  :         6421.83409693 Eh          174746.98974 eV
Electronic Energy  :       -11334.83559126 Eh         -308436.55705 eV
One Electron Energy:       -19590.49932145 Eh         -533084.58803 eV
```

```
Two Electron Energy:         8255.66373018 Eh          224648.03098 eV

Virial components:
Potential Energy    :        -9816.60079460 Eh         -267123.28791 eV
Kinetic Energy      :         4903.59930027 Eh          133433.72060 eV
Virial Ratio        :            2.00191741


DFT components:
N(Alpha)            :       162.000108015896 electrons
N(Beta)             :       160.000112083818 electrons
N(Total)            :       322.000220099714 electrons
E(X)                :      -280.253584159937 Eh
E(C)                :       -15.103060772024 Eh
E(XC)               :      -295.356644931961 Eh
DFET-embed. en.     :         0.000000000000 Eh

--------------------------------------------
MULLIKEN ATOMIC CHARGES AND SPIN POPULATIONS
--------------------------------------------
   0 Cu:    0.450368    0.545780    Cu1
   1 Cu:    0.496849    0.522333    Cu2
   2 O :   -0.491274    0.121572    O1
   3 O :   -0.530986    0.145431    O2
   4 N :   -0.557317    0.060623    N1
   5 N :    0.374211   -0.018157    N2
   6 N :   -0.392301    0.097265    N3
   7 N :   -0.591461    0.070771    N6
   8 N :    0.440541   -0.013934    N7
   9 N :   -0.328241    0.080088    N8
  10 N :   -0.510876    0.090330    N9
  11 N :   -0.472247    0.085439    N4
  12 N :   -0.906185    0.085682    N5
  13 N :   -1.039279    0.092703    N10
```

Singlet state:

```
----------------
TOTAL SCF ENERGY
----------------

Total Energy        :        -4913.00149625 Eh         -133689.56736 eV

Components:
Nuclear Repulsion   :         6421.83409693 Eh          174746.98974 eV
Electronic Energy   :       -11334.83559319 Eh         -308436.55710 eV
One Electron Energy:        -19590.49852793 Eh         -533084.56644 eV
Two Electron Energy:          8255.66293475 Eh          224648.00934 eV

Virial components:
Potential Energy    :        -9816.60071413 Eh         -267123.28572 eV
Kinetic Energy      :         4903.59921787 Eh          133433.71836 eV
Virial Ratio        :            2.00191742


DFT components:
N(Alpha)            :       161.000112153451 electrons
N(Beta)             :       161.000107997287 electrons
N(Total)            :       322.000220150738 electrons
E(X)                :      -280.253565844492 Eh
E(C)                :       -15.103058884178 Eh
```

```
E(XC)               :     -295.356624728670 Eh
DFET-embed. en.     :        0.000000000000 Eh

---------------------------------------------
MULLIKEN ATOMIC CHARGES AND SPIN POPULATIONS
---------------------------------------------
   0 Cu:    0.450354    0.546936
   1 Cu:    0.496876   -0.523120
   2 O :   -0.491283    0.121737
   3 O :   -0.530945   -0.145465
   4 N :   -0.557323    0.060324
   5 N :    0.374209   -0.018040
   6 N :   -0.392280    0.097800
   7 N :   -0.591481   -0.066908
   8 N :    0.440503    0.012936
   9 N :   -0.328339   -0.082645
  10 N :   -0.510867   -0.090334
  11 N :   -0.472244    0.084550
  12 N :   -0.906206    0.085670
  13 N :   -1.039261   -0.092740

-------------------------------------------
BROKEN SYMMETRY MAGNETIC COUPLING ANALYSIS
-------------------------------------------

S(High-Spin)       =   1.0
<S**2>(High-Spin) =   2.0091
<S**2>(BrokenSym) =   1.0091
E(High-Spin)       = -4913.001494 Eh
E(BrokenSym)       = -4913.001496 Eh
E(High-Spin)-E(BrokenSym)= 0.0001 eV     0.422 cm**-1 (ANTIFERROMAGNETIC coupling)

          ----------------------------------------------------------
          | Spin-Hamiltonian Analysis based on H(HDvV)= -2J*SA*SB |
    -------                                                  -----------
    | J(1) =      -0.42 cm**-1    (from -(E[HS]-E[BS])/Smax**2)              |
    | J(2) =      -0.21 cm**-1    (from -(E[HS]-E[BS])/(Smax*(Smax+1))       |
    | J(3) =      -0.42 cm**-1    (from -(E[HS]-E[BS])/(<S**2>HS-<S**2>BS)) |
    --------------------------------------------------------------------------
```

Listing S7. Selected output of the ORCA DFT broken symmetry calculations of cation of $[Cu_2(N_3)(L^c)_2](ClO_4)_3$ (refcode JUDNED, Figure S4). The numberings of the atoms, corresponding to X-ray structures, are shows in red colour (for triplet state).

Triplet state:

```
----------------
TOTAL SCF ENERGY
----------------

Total Energy       :        -5864.62124308 Eh         -159584.45714 eV

Components:
Nuclear Repulsion  :        12525.53342885 Eh          340837.09242 eV
Electronic Energy  :       -18390.15467193 Eh         -500421.54956 eV
One Electron Energy:       -32921.27971782 Eh         -895833.56442 eV
Two Electron Energy:        14531.12504589 Eh          395412.01486 eV

Virial components:
```

```
Potential Energy    :       -11710.66113361 Eh         -318663.28997 eV
Kinetic Energy      :         5846.03989053 Eh          159078.83283 eV
Virial Ratio        :            2.00317845


DFT components:
N(Alpha)            :      232.999572492644 electrons
N(Beta)             :      230.999571413134 electrons
N(Total)            :      463.999143905778 electrons
E(X)                :     -380.517250873031 Eh
E(C)                :      -21.568533633494 Eh
E(XC)               :     -402.085784506525 Eh
DFET-embed. en.     :        0.000000000000 Eh

--------------------------------------------
MULLIKEN ATOMIC CHARGES AND SPIN POPULATIONS
--------------------------------------------
   0 Cu:    0.188817    0.621259    Cu1
   1 Cu:    0.194687    0.623918    Cu2
   2 N :   -0.493340    0.138279    N1
   3 N :    0.480586   -0.030747    N2
   4 N :   -0.199956    0.120699    N3
   5 N :   -0.193930    0.076225    N4
   6 N :   -0.196781    0.091276    N6
   7 N :   -0.194955    0.072650    N8
   8 N :   -0.214488    0.091853    N10
   9 N :   -0.262811    0.005698    N12
  10 N :   -0.244225    0.089890    N14
  11 N :   -0.234495    0.006670    N16
  12 N :   -0.223861    0.087225    N18
```

Singlet state:

```
----------------
TOTAL SCF ENERGY
----------------

Total Energy        :        -5864.62221721 Eh         -159584.48365 eV

Components:
Nuclear Repulsion   :        12525.53342885 Eh          340837.09242 eV
Electronic Energy   :       -18390.15564607 Eh         -500421.57607 eV
One Electron Energy:        -32921.34106164 Eh         -895835.23367 eV
Two Electron Energy:         14531.18541557 Eh          395413.65760 eV

Virial components:
Potential Energy    :       -11710.66394492 Eh         -318663.36647 eV
Kinetic Energy      :         5846.04172771 Eh          159078.88283 eV
Virial Ratio        :            2.00317830


DFT components:
N(Alpha)            :      231.999575187813 electrons
N(Beta)             :      231.999571306257 electrons
N(Total)            :      463.999146494070 electrons
E(X)                :     -380.517242606390 Eh
E(C)                :      -21.568799221134 Eh
E(XC)               :     -402.086041827524 Eh
DFET-embed. en.     :        0.000000000000 Eh

--------------------------------------------
```

```
MULLIKEN ATOMIC CHARGES AND SPIN POPULATIONS
--------------------------------------------
   0 Cu:    0.187254    0.609588
   1 Cu:    0.193215   -0.611878
   2 N :   -0.486797   -0.001584
   3 N :    0.482775    0.000411
   4 N :   -0.193543   -0.000865
   5 N :   -0.193997    0.075997
   6 N :   -0.197553    0.090003
   7 N :   -0.195065   -0.072453
   8 N :   -0.215447   -0.090456
   9 N :   -0.264073    0.003406
  10 N :   -0.245390    0.088604
  11 N :   -0.235935   -0.004156
  12 N :   -0.225129   -0.085822

BROKEN SYMMETRY MAGNETIC COUPLING ANALYSIS
------------------------------------------

S(High-Spin)       =   1.0
<S**2>(High-Spin) =   2.0074
<S**2>(BrokenSym) =   0.9761
E(High-Spin)       = -5864.621243 Eh
E(BrokenSym)       = -5864.622217 Eh
E(High-Spin)-E(BrokenSym)= 0.0265 eV    213.778 cm**-1 (ANTIFERROMAGNETIC
coupling)

          ---------------------------------------------------------
          | Spin-Hamiltonian Analysis based on H(HDvV)= -2J*SA*SB |
    -------                                                        -----------
    | J(1) =    -213.78 cm**-1    (from -(E[HS]-E[BS])/Smax**2)               |
    | J(2) =    -106.89 cm**-1    (from -(E[HS]-E[BS])/(Smax*(Smax+1))        |
    | J(3) =    -207.28 cm**-1    (from -(E[HS]-E[BS])/(<S**2>HS-<S**2>BS)) |
    --------------------------------------------------------------------------
```
